# Complementary PLS and KNN algorithms for improved 3D-QSDAR consensus modeling of AhR binding

**DOI:** 10.1186/1758-2946-5-47

**Published:** 2013-11-21

**Authors:** Svetoslav H Slavov, Bruce A Pearce, Dan A Buzatu, Jon G Wilkes, Richard D Beger

**Affiliations:** 1 Division of Systems Biology, National Center for Toxicological Research, US Food and Drug Administration, 3900 NCTR Road, Jefferson, AR 72079, USA

**Keywords:** QSAR, Molecular descriptors, Quantitative spectral data-activity relationship (3D-QSDAR), Estrogen receptor binding, Molecular modeling

## Abstract

Multiple validation techniques (Y-scrambling, complete training/test set randomization, determination of the dependence of R^2^_test_ on the number of randomization cycles, etc.) aimed to improve the reliability of the modeling process were utilized and their effect on the statistical parameters of the models was evaluated. A consensus partial least squares (PLS)-similarity based k-nearest neighbors (KNN) model utilizing 3D-SDAR (three dimensional spectral data-activity relationship) fingerprint descriptors for prediction of the log(1/EC_50_) values of a dataset of 94 aryl hydrocarbon receptor binders was developed. This consensus model was constructed from a PLS model utilizing *10 ppm x 10 ppm x 0.5 Å* bins and 7 latent variables (R^2^_test_ of 0.617), and a KNN model using *2 ppm x 2 ppm x 0.5 Å* bins and 6 neighbors (R^2^_test_ of 0.622). Compared to individual models, improvement in predictive performance of approximately 10.5% (R^2^_test_ of 0.685) was observed. Further experiments indicated that this improvement is likely an outcome of the complementarity of the information contained in 3D-SDAR matrices of different granularity. For similarly sized data sets of Aryl hydrocarbon (AhR) binders the consensus KNN and PLS models compare favorably to earlier reports. The ability of 3D-QSDAR (three dimensional quantitative spectral data-activity relationship) to provide structural interpretation was illustrated by a projection of the most frequently occurring bins on the standard coordinate space, thus allowing identification of structural features related to toxicity.

## Background

During the past decade, the application of consensus modeling to various QSAR related problems has been explored [1-3]. Early QSARs often relied on single models, which under certain circumstances were prone to arbitrary overestimation of the contribution of given structural features at the expense of others that were suppressed or ignored. To mitigate such risks consensus models based on a multitude of individual models can be advantageously used. Reports of improved performance of consensus models [4-6] or its lack thereof [7] have been published.

Recently, our group introduced the concept of a robust 3D-QSDAR approach [8]. 3D-QSDAR utilizes unique fingerprints constructed from pairs of ^13^C chemical shifts augmented with their corresponding inter-atomic distances. The proposed 3D-QSDAR methodology was designed in accordance with the Organization for Economic Cooperation and Development (OECD) principles [9]: it provided several levels of validation, thus assuring models would be both reliable and interpretable. In our earlier work [8] an automated partial least squares (PLS) algorithm was used to process data from regularly tessellated 3D-SDAR fingerprints and to derive averaged (composite model) predictions from 100 randomized training/hold-out test set pairs. A technique [10] based on the standard deviation of the experimental data was employed to determine a “realistic” upper bound for coefficient of determination. A Y-scrambling procedure [11,12] assessed the probability of generating seemingly “good” models by chance.

However, the above described modeling procedure employed a single data processing algorithm, namely PLS. As a step forward, experiments designed to explore the likelihood of building improved consensus models combining predictions generated by conceptually unrelated algorithms operating on 3D-SDAR matrices of different granularity were conceived. A KNN algorithm intended to supplement PLS by capturing complementary aspects of the structure-activity relationship was devised. It was hypothesized that the improvement of performance in consensus modeling should depend on the degree of orthogonality of the predictions produced by the individual models. Beyond the accuracy of biological data the inherent information content of a given descriptor pool was thought of as a factor limiting the improvement of R^2^_test_ in consensus modeling. In other words, regardless of data processing algorithm the maximum achievable R^2^ for a hold-out test set would be limited by the descriptors’ ability to depict specific aspects of the molecular structure directly related to the observed effect.

In this work, the effect of data processing algorithms on the quality of prediction and the ability of 3D-QSDAR to provide a meaningful structural interpretation was tested on a dataset of 94 PCBs, PHDDs and PCDFs binding the aryl hydrocarbon receptor (AhR). Similar datasets were extensively modeled (Table 1 summarizes these results) and the structural features affecting binding are well known. This allowed quantitative and qualitative comparison of these earlier reports with the results carried out using the 3D-QSDAR methodology.

**Table 1:** Summary of QSARs published since year 2000

**Chemical class**	**Endpoint**	**Dataset size**	Data processing algorithm*	**Descriptor type**	**Statistical parameters**	**Reference**
PCBs, PCDDs and PCDFs	logEC_50_	52	MLR	^13^C-NMR	R^2^ = 0.85; q^2^ = 0.71	[13]
PCBs, PCDDs and PCDFs	logEC_50_	52	MLR	^13^C-NMR, atom-to-atom distances	R^2^ = 0.85; q^2^ = 0.52	[14]
PCDFs	log(1/EC_50_)	33	MLR	Quantum mechanical; logP	R^2^ = 0.720; s = 0.723	[15]
PCDFs	log(1/EC_50_)	34	MLR	Quantum mechanical	R^2^ = 0.747; R^2^_adj_ = 0.669; q^2^ = 0.572	[16]
PCDDs and PCDFs	log(1/EC_50_)	90	PLS	CoMFA - 10 latent variables	R^2^ = 0.838; q^2^ = 0.624; SEP = 0.903	[17]
PCDFs	log(1/EC_50_)	34	MLR	Quantum mechanical	R^2^ = 0.863; R^2^_adj_ = 0.839; q^2^ = 0.807; SE = 0.558 ; F = 35.389	[18]
PCDDs	log(1/EC_50_)	47	MLR	Quantum mechanical	R^2^ = 0.729; R^2^_adj_ = 0.703; SE = 0.797; F = 28.269	[19]
PHDDs	log(1/EC_50_)	25	MLR	Quantum mechanical	R^2^ = 0.768; R^2^_adj_ = 0.721; q^2^ = 0.635; S.E. = 0.762; F = 16.529	[20]
PHDDs	log(1/EC_50_)	25	MLR	WHIM	R^2^ = 0.915; R^2^_adj_ = 0.902; q^2^ = 0.880; S.E. = 0.451; F = 75.032	[20]
PCDDs and PCDFs	log(1/EC_50_)	60	MLR	Quantum mechanical	R^2^ = 0.687; R^2^_adj_ = 0.686; q^2^ = 0.603; S.E. = 0.870	[21]

*MLR - Multiple Linear Regression; PLS - Partial Least Squares.

### Dataset

A dataset compiled by Mekenyan et al. [22] containing the experimental log(1/EC_50_) values of 94 persistent organic pollutants inhibiting AhR was used for the purpose of this work. Here, EC_50_ is defined as the concentration of the test chemical necessary to reduce the specific binding of a radiolabeled 2,3,7,8-tetrachlorodibenzo-*p*-dioxin to 50% of its maximum value in the absence of competitor. This dataset consists of three distinct chemical classes: i) polychlorinated biphenyls (PCBs); ii) polyhalogenated dibenzo-*p*-dioxins (PHDDs) and iii) polychlorinated dibenzofurans (PCDFs), shown in Table 2. Since we were unable to locate sources reporting the experimental errors of the EC_50_ data for the current dataset, a work by Long et al. [23] listing the absolute errors of 7 PCDF congeners was used to evaluate the quality of data. Under the assumption that for similar compounds and experimental settings the average relative experimental error would vary insignificantly, based on the above report at least ~17% error in the EC_50_ data should be expected. However, since Mekenyan et al. [22] compiled their dataset from various sources, a negative impact on the accuracy of data which would further lower the “realistic” R^2^[10] should be anticipated.

**Table 2:** **AhR binders and their experimental and predicted log(1/EC**_**50**_**)**

**Chemical name**	**Experimental log(1/EC**_**50**_**)**	**Predicted log(1/EC**_**50**_**)**				
		**2 ppm × 2 ppm × 0.5Å**		**10 ppm × 10 ppm × 0.5Å**		***PLS-KNN *** **consensus from II and III**
		**I*PLS***	**II*KNN***	**III*PLS***	**IV*KNN***	
3,3',4,4'-Tetrachlorobiphenyl	6.15	6.02	5.50	6.49	5.75	6.00
2,3,4,4'-Tetrachlorobiphenyl	4.55	5.27	5.35	5.15	5.28	5.25
3,3',4,4',5-Pentachlorobiphenyl	6.89	5.06	5.11	5.96	5.63	5.54
2',3,4,4',5-Pentachlorobiphenyl	4.85	4.77	5.26	4.23	5.11	4.75
2,3,3',4,4'-Pentachlorobiphenyl	5.37	5.59	5.64	5.07	5.38	5.36
2,3',4,4',5-Pentachlorobiphenyl	5.04	5.47	5.47	4.74	5.29	5.11
2,3,4,4',5-Pentachlorobiphenyl	5.39	4.81	4.78	5.53	5.14	5.16
2,3,3',4,4',5-Hexachlorobiphenyl	5.15	5.33	5.22	5.61	5.19	5.42
2,3',4,4',5,5'-Hexachlorobiphenyl	4.80	5.16	5.41	4.80	5.36	5.11
2,3,3'4,4',5'-Hexachlorobiphenyl	5.33	5.23	5.12	5.07	5.37	5.10
2,2',4,4'-Tetrachlorobiphenyl	3.89	5.22	4.83	4.49	4.94	4.66
2,2',4,4'5,5'-Hexachlorobiphenyl	4.10	4.41	5.05	3.50	4.85	4.28
2,3,4,5-Tetrachlorobiphenyl	3.85	5.55	5.20	5.35	5.29	5.28
2,3',4,4',5',6-Hexachlorobiphenyl	4.00	5.44	5.23	4.37	4.90	4.80
4'-Hydroxy-2,3,4,5-tetrachlorobiphenyl	4.05	5.88	5.05	5.07	4.86	5.07
4'-Methyl-2,3,4,5-tetrachlorobiphenyl	4.51	5.21	5.27	5.13	4.86	5.20
4'-Fluoro-2,3,4,5-tetrachlorobiphenyl	4.60	5.13	4.92	4.37	4.67	4.65
4'-Methoxy-2,3,4,5-tetrachlorobiphenyl	4.80	5.35	5.15	4.32	4.74	4.74
4'-Acetyl-2,3,4,5-tetrachlorobiphenyl	5.17	5.00	4.87	4.14	4.98	4.51
4'-Cyano-2,3,4,5-tetrachlorobiphenyl	5.27	5.48	5.05	4.29	4.78	4.67
4'-Ethyl-2,3,4,5-tetrachlorobiphenyl	5.46	5.13	5.06	4.50	4.82	4.78
4'-Bromo-2,3,4,5-tetrachlorobiphenyl	5.60	5.42	5.34	5.27	5.51	5.31
4'-Iodo-2,3,4,5-tetrachlorobiphenyl	5.82	5.53	5.16	5.88	5.84	5.52
4'-isopropyl-2,3,4,5-tetrachlorobiphenyl	5.89	5.77	5.45	5.07	4.75	5.26
4'-Trifluromethyl-2,3,4,5-tetrachlorobiphenyl	6.43	5.42	5.25	4.46	4.80	4.86
3'-Nitro-2,3,4,5-tetrachlorobiphenyl	4.85	5.51	5.27	5.07	4.75	5.17
4'-N-Acetylamino-2,3,4,5-tetrachlorobiphenyl	5.09	5.26	4.87	5.09	4.96	4.98
4'-Phenyl-2,3,4,5-tetrachlorobiphenyl	5.18	4.74	5.03	4.69	5.01	4.86
4'-t-Butyl-2,3,4,5-tetrachlorobiphenyl	5.17	5.12	5.34	4.71	4.89	5.03
4'-n-Butyl-2,3,4,5-tetrachlorobiphenyl	5.13	5.12	5.13	5.44	4.93	5.29
2,3,7,8-Tetrachlorodibenzo-p-dioxin	8.00	8.27	7.66	7.10	7.28	7.38
1,2,3,7,8-Pentachlorodibenzo-p-dioxin	7.10	6.10	6.73	6.43	5.99	6.58
2,3,6,7-Tetrachlorodibenzo-p-dioxin	6.80	6.56	6.76	5.92	5.96	6.34
2,3,6-Trichlorodibenzo-p-dioxin	6.66	6.31	6.67	5.85	5.90	6.26
1,2,3,4,7,8-Hexachlorodibenzo-p-dioxin	6.55	5.83	6.10	5.84	5.69	5.97
1,3,7,8-Tetrachlorodibenzo-p-dioxin	6.10	6.22	6.68	6.03	6.12	6.36
1,2,4,7,8-Pentachlorodibenzo-p-dioxin	5.96	5.99	6.46	5.41	5.86	5.94
1,2,3,4-Tetrachlorodibenzo-p-dioxin	5.89	4.39	5.44	5.96	5.87	5.70
2,3,7-Trichlorodibenzo-p-dioxin	7.15	6.72	6.84	6.69	7.37	6.77
2,8-Dichlorodibenzo-p-dioxin	5.50	5.73	6.04	7.83	7.94	6.94
1,2,3,4,7-Pentachlorodibenzo-p-dioxin	5.19	5.68	6.02	5.69	5.95	5.86
1,2,4-Trichlorodibenzo-p-dioxin	4.89	5.46	5.90	6.12	5.99	6.01
1,2,3,4,6,7,8,9-octachlorodibenzo-p-dioxin	5.00	6.78	7.76	4.77	5.74	6.27
1-Chlorodibenzo-p-dioxin	4.00	5.97	6.09	6.44	6.54	6.28
2,3,7,8-Tetra bromodibenzo-p-dioxin	8.82	9.29	8.61	9.86	8.43	9.24
2,3-Dibromo-7,8-dichlorodibenzo-p-dioxin	8.83	8.56	8.43	8.55	8.15	8.49
2,8-Dibromo-3,7-dichlorodibenzo-p-dioxin	9.35	7.54	7.86	6.87	7.06	7.37
2-Bromo-3,7,8-trichlorodibenzo-p-dioxin	7.94	8.31	8.05	7.26	7.40	7.66
1,3,7,8,9-Pentabromodibenzo-p-dioxin	7.03	7.25	7.99	7.53	8.29	7.76
1,3,7,8,-Tetrabromodibenzo-p-dioxin	8.70	7.38	8.51	8.22	8.48	8.37
1,2,4,7,8-Pentabromodibenzo-p-dioxin	7.77	7.31	8.06	9.20	8.24	8.63
1,2,3,7,8-Pentabromodibenzo-p-dioxin	8.18	8.31	8.65	8.40	8.57	8.53
2,3,7-Tribromodibenzo-p-dioxin	8.93	8.10	8.40	8.23	8.42	8.32
2,7-Dibromodibenzo-p-dioxin	7.81	7.48	7.36	7.07	8.06	7.22
2-Bromodibenzo-p-dioxin	6.53	6.67	7.03	8.22	7.73	7.63
2-Chlorodibenzofuran	3.55	3.94	4.48	3.76	3.78	4.12
3-Chlorodibenzofuran	4.38	5.13	5.01	5.75	5.89	5.38
4-Chlorodibenzofuran	3.00	5.20	4.54	4.80	5.37	4.67
2,3-Dichlorodibenzofuran	5.33	5.29	4.77	5.68	5.71	5.23
2,6-Dichlorodibenzofuran	3.61	5.03	4.85	3.50	4.14	4.18
2,8-Dichlorodibenzofuran	3.59	4.21	4.77	3.76	3.88	4.27
1,3,6-Trichlorodibenzofuran	5.36	6.28	6.21	5.70	5.57	5.96
1,3,8-Trichlorodibenzofuran	4.07	5.80	5.82	5.28	5.40	5.55
2,3,4-Trichlorodibenzofuran	4.72	6.78	5.80	5.73	5.83	5.77
2,3,8-Trichlorodibenzofuran	6.00	5.58	5.07	5.63	5.59	5.35
2,6,7 -Trichlorodibenzofuran	6.35	5.64	5.29	5.38	4.98	5.34
2,3,4,6-Tetrachlorodibenzofuran	6.46	5.95	5.86	6.68	5.56	6.27
2,3,4,8-Tetrachlorodibenzofuran	6.70	6.19	5.84	5.55	5.38	5.70
1,3,6,8-Tetrachlorodibenzofuran	6.66	5.63	5.52	6.36	5.92	5.94
2,3,7,8-Tetrachlorodibenzofuran	7.39	6.96	6.54	7.18	6.84	6.86
1,2,4,8-Tetrachlorodibenzofuran	5.00	5.16	5.32	4.19	4.90	4.76
1,2,4,6,7-Pentachlorodibenzofuran	7.17	5.65	5.50	5.82	5.54	5.66
1,2,4,7,9-Pentachlorodibenzofuran	4.70	6.82	6.34	5.22	5.40	5.78
1,2,3,4,8-Pentachlorodibenzofuran	6.92	6.42	5.74	5.49	5.21	5.62
1,2,3,7,8-Pentachlorodibenzofuran	7.13	7.03	6.56	6.96	7.19	6.76
1,2,4,7,8-Pentachlorodibenzofuran	5.89	5.94	5.57	6.32	5.94	5.95
2,3,4,7,8-Pentachlorodibenzofuran	7.82	6.42	6.42	7.08	6.80	6.75
1,2,3,4,7,8-Hexachlorodibenzofuran	6.64	6.61	6.06	7.22	6.95	6.64
1,2,3,6,7,8-Hexachlorodibenzofuran	6.57	7.22	6.78	6.67	6.47	6.73
1,2,4,6,7,8-Hexachlorodibenzofuran	5.08	6.58	5.83	6.53	5.70	6.18
2,3,4,6,7,8-Hexachlorodibenzofuran	7.33	7.93	6.85	7.73	6.60	7.29
2,3,6,8-Tetrachlorodibenzofuran	6.66	5.39	5.23	5.58	5.42	5.41
1,2,3,6-Tetrachlorodibenzofuran	6.46	4.93	5.36	6.17	5.85	5.77
1,2,3,7-Tetrachlorodibenzofuran	6.96	6.93	6.57	7.00	7.22	6.79
1,3,4,7,8-Pentachlorodibenzofuran	6.70	6.82	6.59	6.60	6.53	6.60
2,3,4,7,9-Pentachlorodibenzofuran	6.70	6.54	6.34	7.29	6.99	6.82
1,2,3,7,9-Pentachlorodibenzofuran	6.40	6.32	6.40	6.69	6.94	6.55
H	3.00	3.53	4.46	3.98	3.95	4.22
2,3,4,7-Tetrachlorodibenzofuran	7.60	6.08	6.44	6.37	6.29	6.41
1,2,3,7-Tetrachlorodibenzofuran	6.96	6.97	6.59	7.00	7.17	6.80
1,3,4,7,8-Pentachlorodibenzofuran	6.70	6.84	6.58	6.62	6.52	6.60
2,3,4,7,9-Pentachlorodibenzofuran	6.70	6.52	6.36	7.23	6.96	6.80
1,2,3,7,9-Pentachlorodibenzofuran	6.40	6.38	6.41	6.68	6.94	6.55
1,2,4,6,8-Pentachlorodibenzofuran	5.51	5.81	5.61	3.30	4.80	4.46

## Methods

### Conventions

Several layers of complexity related to the utilized modeling procedures were introduced in this manuscript and these require clarification. To avoid ambiguity, models utilizing the same algorithm (either PLS or KNN) operating on an individual 3D-SDAR data matrix by generating multiple randomized training/test subset pairs later combined to form a single model will be referred to as “composite models”. Models averaging the predictions from two (or eventually more) composite models will be referred to as “consensus models”. The term “individual models” is used interchangeably to denote either the individual PLS or KNN models forming the “consensus model” or the individual randomized training/test subset models resulting in a “composite model”. However, its specific meaning would be determined through its contextual use. The term “matching training/test subset pairs” indicates complementary training and test subset pairs processed by different algorithms, but composed of the same subsets of compounds.

### Molecular conformation

In its current implementation, 3D-QSDAR does not employ docking or alignment algorithms, nor does it use X-ray structures to achieve more consistent geometries of the molecules constituting the dataset. This choice widens its applicability to datasets of compounds with unknown, multiple, or no specific targets and in the absence of knowledge about the binding site and its conformational requirements. For the purpose of reproducibility, however, the conformation at the global minimum of the potential energy surface was used. It has to be acknowledged that, while this conformation is the most energetically stable, it may not be the one assumed during solvent interaction or upon binding with a macromolecule [24].

To find the lowest energy conformers of all PCBs (the PHDDs and PCDFs have no rotatable bonds) a conformational search analysis was performed in HyperChem 8.0 [25]. An AMBER force field [26] and a random walks search method with an acceptance energy criterion of 6 kcal/mol were used. At the final stage of optimization all PCBs, PHDDs and PCDFs were optimized by employing a semi-empirical Austin Model 1 (AM1) Hamiltonian with a root-mean-square gradient of 0.01 kcal/Å × mol.

### 3D-QSDAR descriptor calculations

**Figure 1:**
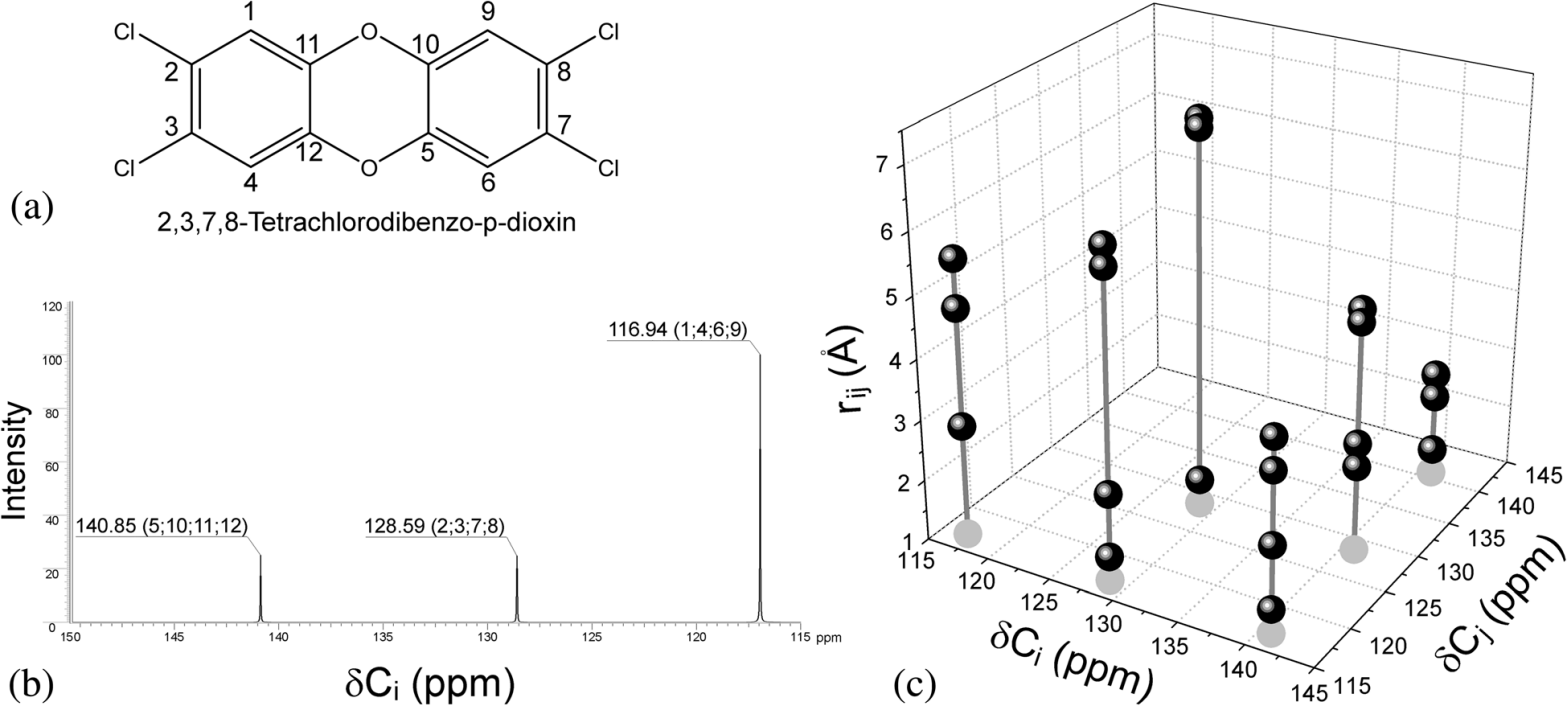
**(a) structure of 2,3,7,8-tetrachlorodibenzo-*p*-dioxin; (b) **^**13**^**C NMR spectra of 2,3,7,8-tetrachlorodibenzo-*p*-dioxin; (c) 3D fingerprint of 2,3,7,8-tetrachlorodibenzo-*p*-dioxin; The gray circles representing the shadows of the fingerprint elements in the *XY*-plane and the drop lines are shown to indicate better the elements’ positions in the 3D-SDAR abstract space.**

The final refined geometries of all 94 AhR binders together with their respective ^13^C chemical shifts simulated by ACD/NMR Predictor version 12.0 [27] were used to generate unique 3D-fingerprints representing each compound in the abstract 3D-SDAR space [8]. This space is defined by three orthogonal coordinates, with the chemical shifts of atoms C_i_ and C_j_ on axes *X* and *Y*, respectively, and the distance between them on the *Z* axis. This concept is illustrated in Figure 1, which shows the structure and the ^13^C-NMR spectrum of 2,3,7,8-tetrachlorodibenzo-*p*-dioxin (Figures 1a and 1b) and its corresponding 3D-SDAR fingerprint (Figure 1c). For example, in Figure 1c the coordinates of the topmost left fingerprint element are defined by the chemical shifts of atoms 1 and 6 (116.94 ppm) and the distance between them (5.52 Å). Due to the D_2h_ symmetry of the 2,3,7,8-tetrachlorodibenzo-*p*-dioxin molecule, the position of this fingerprint element in the 3D-SDAR space coincides with the one representing atoms 4 and 9. The remaining fingerprint elements are constructed in a similar manner.

Because the units of length on each axis are not identical, *X*, *Y* and *Z* do not form a Cartesian coordinate system. Since the number of carbon atoms in a molecule (*N*_*C*_) determines uniquely the number of elements in a fingerprint (*N*_*C*_(*N*_*C*_ - *1*)/2), each of the 94 AhR binders will be represented by at least 66 such fingerprint elements in the 3D-SDAR space. This 3D-SDAR space was further tessellated using regular grids to form bins ranging in size from *2 ppm x 2 ppm x 0.5 Å* to *20 ppm x 20 ppm x 2.5 Å* (i.e. incremental steps of 0.5 Å on the Z-axis and 2 ppm on the chemical shifts plane *XY* were used). As a result, 50 regular grids of different granularity were generated. A procedure performed separately on each of the 50 grids counted the number of fingerprint elements of a molecule belonging to a given bin (i.e., bin occupancy) and stored these values as row vectors in *m* x *n* matrices. Here *m* represents the number of compounds in the dataset, whereas *n* represents the number of occupied bins.

### Determination of the optimal number of randomization cycles

**Figure 2:**
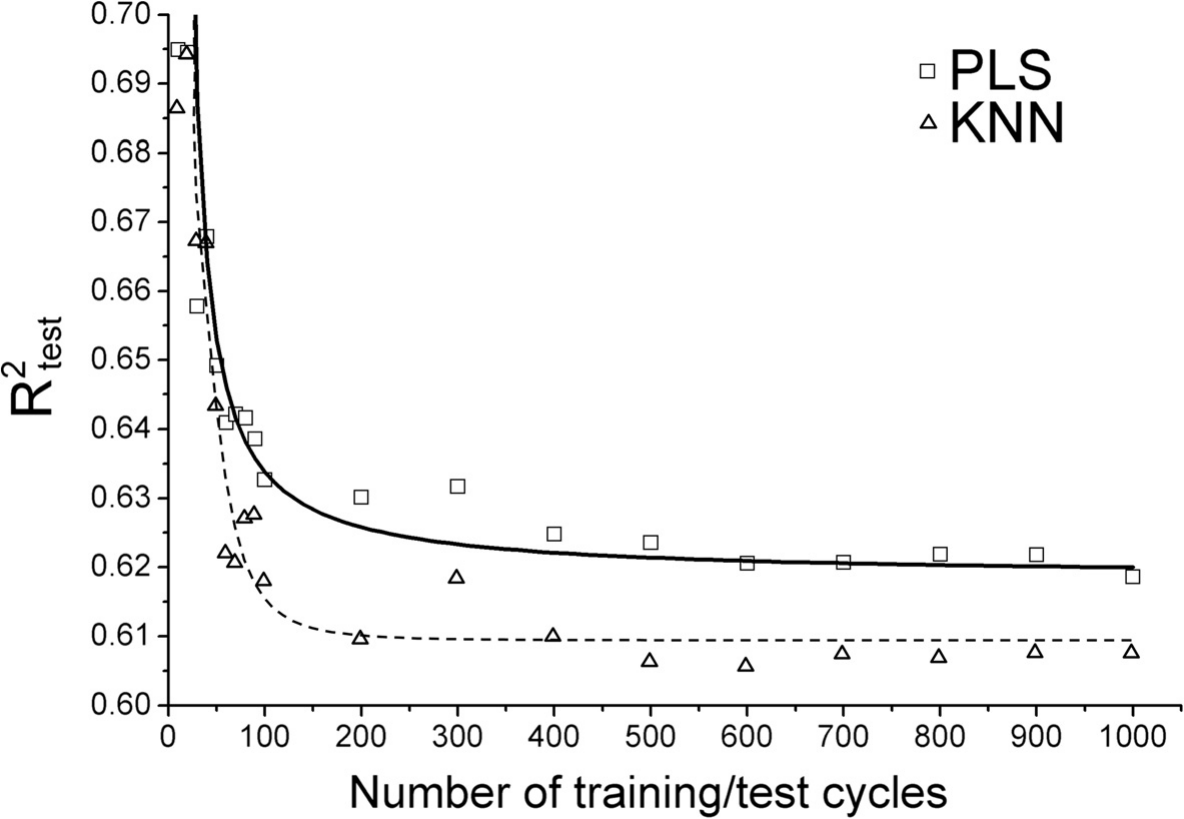
Average predictive performance of the PLS and KNN models as a function of the number of training/test cycles.

Experiments aimed at the determination of the optimal number of training/test subset randomization cycles necessary to achieve an asymptotic convergence of R^2^_test_ (an average of *N* individual R^2^_test_ values, 10 ≤ *N* ≤ 1000) to its “true” value were conducted. As an example, Figure 2 shows the dependence between R^2^_test_ and the number of randomization cycles in case of: i) our best PLS model utilizing *10 ppm x 10 ppm x 0.5 Å* bins and 7 latent variables (LVs) and ii) our best KNN model using *2 ppm x 2 ppm x 0.5 Å* bins and 6 neighbors. Figure 2 indicates that a minimum of 100 randomization cycles would be needed so that the average R^2^_test_ values would converge to their asymptotic values. Therefore, to reduce the computational demand and to avoid reporting overly-optimistic results, 100 randomizations for each of the 50 3D-SDAR data matrices were performed.

### Model building

To explore the ability of different data processing techniques to capture complementary portions of the variance in biological data, two algorithms based on unrelated concepts but operating on descriptor matrices originating from the 3D-QSDAR approach were employed.

i) A SIMPLS based [28] PLS algorithm written in Matlab [29] was employed to process each of the 50 3D-QSDAR data matrices. All descriptors were standardized using the “zscore” Matlab function. As described above, 100 random training/test set pairs were generated and composite (ensemble) PLS models for the training sets, including somewhere between 1 and 10 LVs, were built. These models were then used to predict the log(1/EC_50_) values for the complementary 20% “hold-out” test subsets. At the end, each of the individual 100 R^2^_training_, R^2^_test_ and R^2^_scrambling_ values were recorded and their averages for the composite models were reported. For each of the 50 average models utilizing grids of different granularity the random number generator was initialized in order to recreate the same training/hold-out test sequence (Additional file 1). Due to the specifics of the chosen model-building procedure, the reader should bear in mind that these average reported parameters include contributions from “good” as well as “bad” models (see the results and discussion section).

ii) Alternatively, a KNN algorithm written in Matlab and based on Tanimoto similarity [30] in its generalized vector form, $T\left(A,B\right)=\frac{A.B}{{\left\|A\right\|}^{2}+{\left\|B\right\|}^{2}-A.B}$ was employed. In this equation, *A* and *B* are data objects represented by vectors (originally bit vectors). Thus, the Tanimoto similarity is a dot product of two vectors *A* and *B* (bin occupancy row vectors for a pair of compounds) divided by the squared magnitudes of *A* and *B* minus their dot product. In other words, for compounds sharing common structural features *T* will be closer to 1, otherwise *T* will be closer to 0.

Because *T* is not invariant to standardization, the desire for preservation of its universal nature required use of the original, non-transformed 3D-SDAR descriptor pool. At a constant granularity of the grid this specific choice allowed bijection of *T* - there is one and only one *T* for a given pair of compounds. For a standardized descriptor pool, *T* loses its universal nature by being dependant on the mean and the standard deviation of the descriptors within the training set, and multiple *T*-s between a pair of compounds would exist (i.e., *T* would become a local characteristic of similarity).

These invariant *T* values (calculated for all pairs of compounds) were later used to predict the hold-out test set activities by ranking the compounds from the training set in a descending order of their similarity to each compound from the hold-out test and using *T* of the first *K*-neighbors (1 ≤ *K* ≤ 10) to weight their contributions to activity. Under these experimental settings, both odd and even numbers of neighbors can be used. As with PLS, the KNN validation procedure involved 100 randomized training/hold-out test set pairs recreated by the use of the same random seed.

### Fit and prediction

The majority of QSARs are built for prediction. Hence, parameters such as the coefficient of determination for the training set (R^2^_training_) that measure the fitting ability of a model play only a minor role, typically unrelated to predictive power. Since we are more interested in the behavior of models intended for prediction, our attention was primarily focused on R^2^_test_ and R^2^_scrambling_. More specifically, the behavior of R^2^_test_ was closely followed, whereas R^2^_scrambling_ was monitored only as an indicator of potential chance correlations.

## Results and discussion

### Similarity as a discrimination function

**Figure 3:**
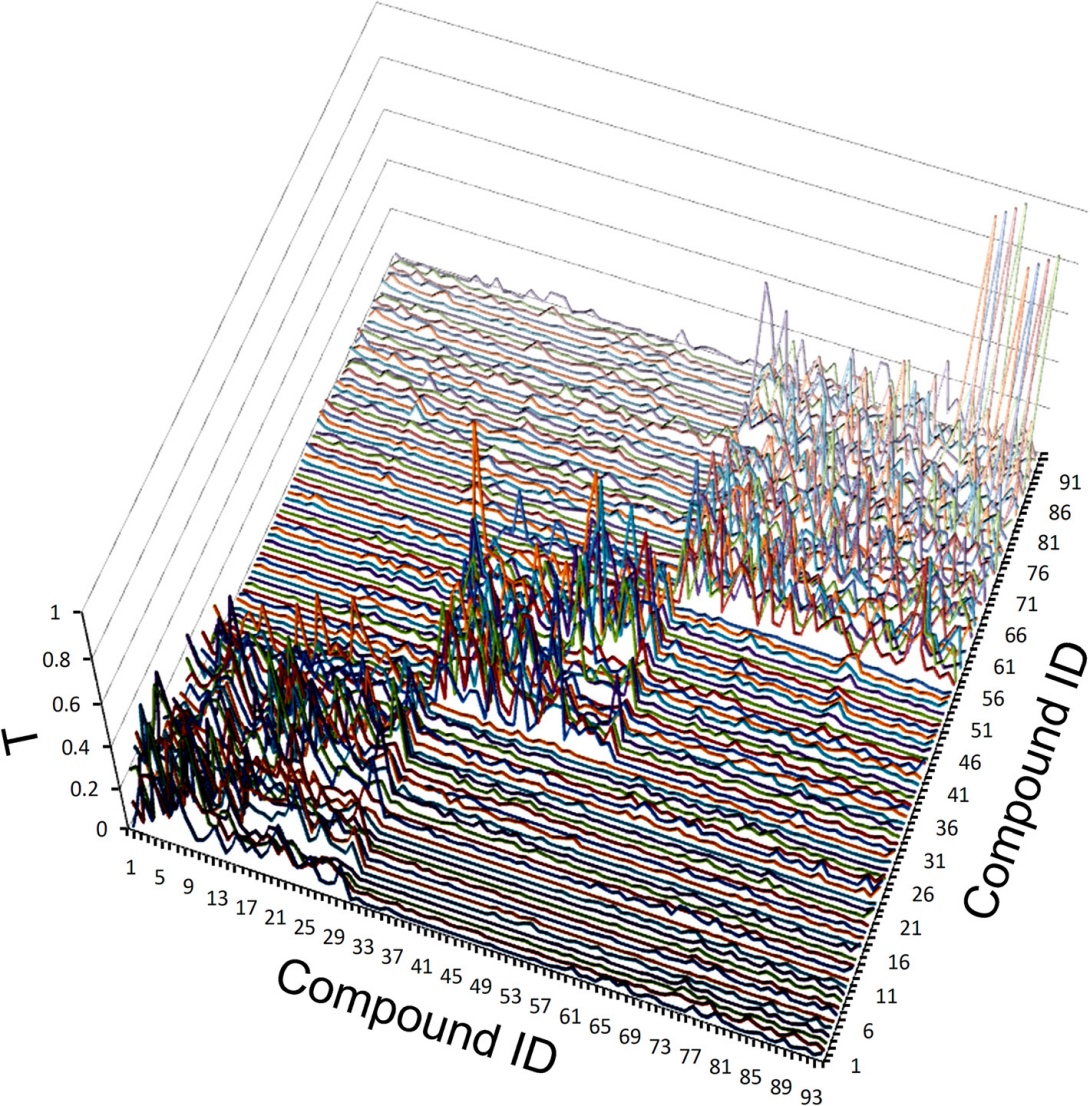
**Tanimoto similarity between pairs of compounds for the AhR dataset using *2 ppm x 2 ppm x 0.5 Å *** **bins.**

The ability of *T* to detect structural similarity and thus structural variations is illustrated in Figure 3 (*2 ppm x 2 ppm x 0.5 Å* bins were used). Three regions of higher similarity are clearly distinguishable: i) compounds with IDs between 1 and 30 are all PCBs; ii) compounds with IDs between 31 and 55 are PHDDs and iii) the remaining 39 compounds are PCDFs. Because *T* is calculated from row vectors, it can be demonstrated that KNN operating on *T* may capture information complementary to that of the respective PLS models (virtually all multivariate methods operate on column vectors). Thus, the degree of orthogonality between PLS and KNN would be one of the factors with an impact on the performance of consensus PLS-KNN models. Another such factor would be the complementarity of the information content specific to 3D-SDAR matrices of different granularity.

### Optimal bin size

**Figure 4:**
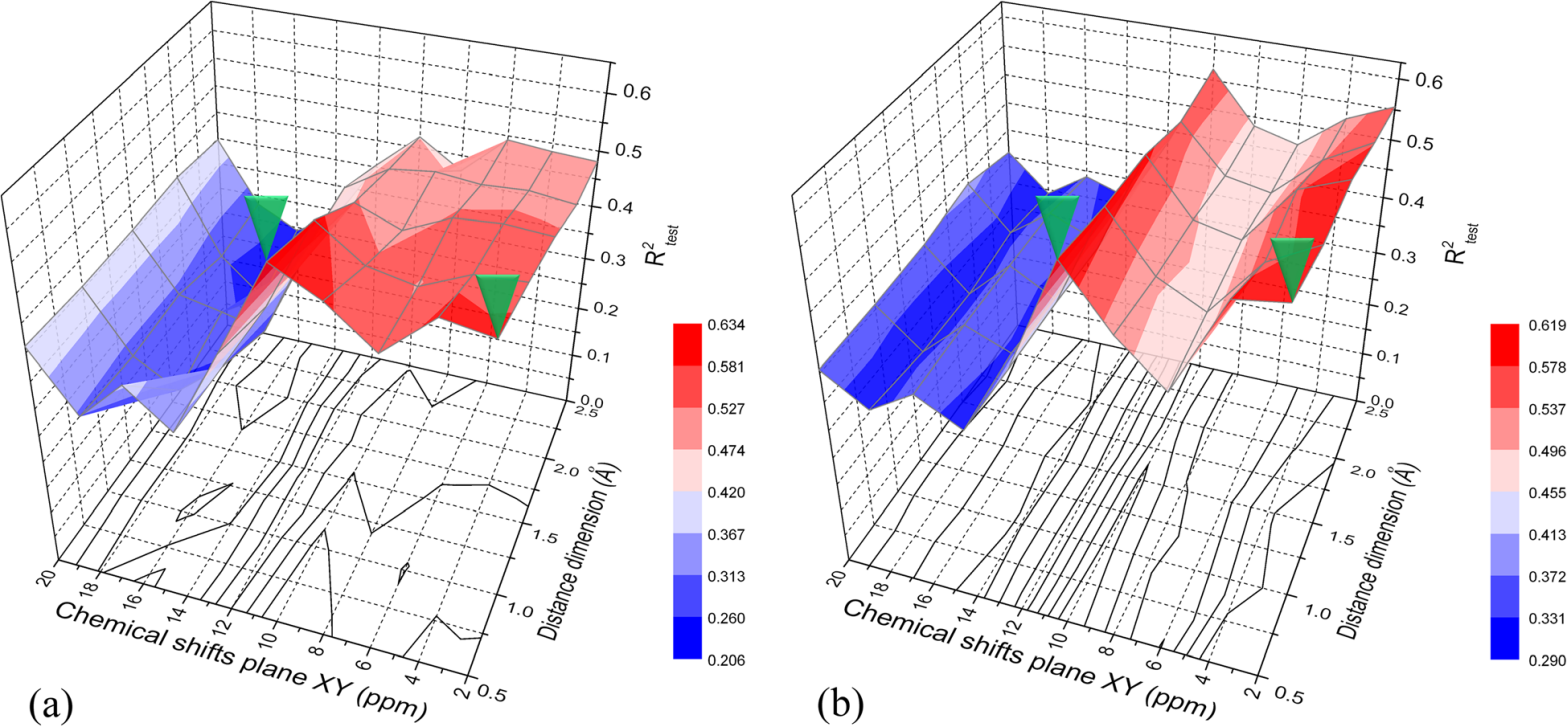
**Average R**^**2**^_**test **_**for the (a) PLS and (b) KNN models as a function of the 3D-bin size.**

As described above a total of 50 PLS and KNN composite models of different granularity (each of which is a result of 100 training/test set combinations) were built. The statistical parameters of these models calculated as an average of their corresponding 100 individual values are shown in Table 3. The predictive power of the PLS and KNN models in terms of R^2^_test_ as a function of the granularity of the 3D-SDAR abstract space is shown on Figure 4.

**Table 3:** Average statistical parameters of the best PLS and KNN models at a given number of LVs and neighbors as a function of the granularity of the 3D-SDAR space

**Bin size**	**Optimal number of LVs**	**Avg. R**^**2**^**test (PLS)**	**Std. R**^**2**^**test (PLS)**	**Avg. R**^**2**^**scr (PLS)**	**Std. R**^**2**^**scr (PLS)**	**Optimal number of neighbors**	**Avg. R**^**2**^**test (KNN)**	**Std. R**^**2**^**test (KNN)**
2 ppm x 2 ppm x 0.5 Å	3	0.591	0.143	0.085	0.103	6	0.618*	0.170
4 ppm x 4 ppm x 0.5 Å	3	0.604	0.142	0.088	0.109	5	0.606	0.146
6 ppm x 6 ppm x 0.5 Å	5	0.532	0.167	0.074	0.097	7	0.453	0.178
8 ppm x 8 ppm x 0.5 Å	5	0.593	0.142	0.097	0.113	6	0.520	0.162
10 ppm x 10 ppm x 0.5 Å	7	0.633*	0.147	0.085	0.113	4	0.612	0.162
12 ppm x 12 ppm x 0.5 Å	3	0.474	0.178	0.105	0.115	9	0.432	0.181
14 ppm x 14 ppm x 0.5 Å	2	0.321	0.193	0.096	0.121	10	0.312	0.179
16 ppm x 16 ppm x 0.5 Å	3	0.383	0.154	0.073	0.090	10	0.353	0.166
18 ppm x 18 ppm x 0.5 Å	2	0.307	0.189	0.077	0.100	10	0.307	0.186
20 ppm x 20 ppm x 0.5 Å	2	0.410	0.178	0.122	0.137	9	0.356	0.180
2 ppm x 2 ppm x 1.0 Å	3	0.567	0.149	0.082	0.095	6	0.599	0.181
4 ppm x 4 ppm x 1.0 Å	3	0.562	0.149	0.081	0.099	3	0.558	0.179
6 ppm x 6 ppm x 1.0 Å	5	0.526	0.164	0.076	0.099	7	0.466	0.178
8 ppm x 8 ppm x 1.0 Å	4	0.542	0.161	0.095	0.116	6	0.504	0.164
10 ppm x 10 ppm x 1.0 Å	6	0.597	0.153	0.086	0.100	4	0.593	0.162
12 ppm x 12 ppm x 1.0 Å	2	0.440	0.176	0.101	0.128	10	0.429	0.182
14 ppm x 14 ppm x 1.0 Å	2	0.315	0.195	0.100	0.125	10	0.327	0.179
16 ppm x 16 ppm x 1.0 Å	5	0.251	0.147	0.069	0.090	10	0.357	0.168
18 ppm x 18 ppm x 1.0 Å	2	0.296	0.189	0.077	0.106	10	0.292	0.185
20 ppm x 20 ppm x 1.0 Å	2	0.405	0.176	0.128	0.137	10	0.358	0.180
2 ppm x 2 ppm x 1.5 Å	3	0.537	0.163	0.074	0.087	5	0.603	0.178
4 ppm x 4 ppm x 1.5 Å	3	0.542	0.151	0.077	0.101	6	0.574	0.160
6 ppm x 6 ppm x 1.5 Å	5	0.536	0.164	0.073	0.112	5	0.481	0.169
8 ppm x 8 ppm x 1.5 Å	8	0.500	0.196	0.090	0.106	9	0.498	0.164
10 ppm x 10 ppm x 1.5 Å	8	0.531	0.180	0.092	0.106	5	0.585	0.166
12 ppm x 12 ppm x 1.5 Å	2	0.440	0.174	0.104	0.132	10	0.421	0.180
14 ppm x 14 ppm x 1.5 Å	8	0.267	0.155	0.073	0.082	10	0.316	0.181
16 ppm x 16 ppm x 1.5 Å	6	0.286	0.147	0.063	0.081	10	0.359	0.169
18 ppm x 18 ppm x 1.5 Å	2	0.302	0.188	0.079	0.111	7	0.291	0.180
20 ppm x 20 ppm x 1.5 Å	2	0.406	0.176	0.121	0.138	10	0.365	0.182
2 ppm x 2 ppm x 2.0 Å	2	0.495	0.177	0.071	0.086	6	0.576	0.180
4 ppm x 4 ppm x 2.0 Å	3	0.504	0.158	0.080	0.102	7	0.535	0.172
6 ppm x 6 ppm x 2.0 Å	5	0.500	0.170	0.071	0.095	6	0.467	0.173
8 ppm x 8 ppm x 2.0 Å	4	0.508	0.159	0.095	0.121	10	0.481	0.169
10 ppm x 10 ppm x 2.0 Å	4	0.498	0.174	0.088	0.105	10	0.557	0.174
12 ppm x 12 ppm x 2.0 Å	3	0.450	0.171	0.102	0.116	10	0.430	0.181
14 ppm x 14 ppm x 2.0 Å	9	0.297	0.156	0.078	0.093	10	0.329	0.186
16 ppm x 16 ppm x 2.0 Å	7	0.207	0.142	0.057	0.075	10	0.359	0.166
18 ppm x 18 ppm x 2.0 Å	2	0.273	0.179	0.070	0.112	10	0.308	0.188
20 ppm x 20 ppm x 2.0 Å	2	0.410	0.174	0.131	0.137	10	0.383	0.179
2 ppm x 2 ppm x 2.5 Å	2	0.481	0.18	0.076	0.087	8	0.555	0.185
4 ppm x 4 ppm x 2.5 Å	3	0.485	0.163	0.079	0.101	7	0.522	0.182
6 ppm x 6 ppm x 2.5 Å	5	0.492	0.165	0.071	0.101	7	0.465	0.175
8 ppm x 8 ppm x 2.5 Å	3	0.422	0.173	0.097	0.122	6	0.485	0.175
10 ppm x 10 ppm x 2.5 Å	10	0.471	0.222	0.072	0.082	3	0.568	0.172
12 ppm x 12 ppm x 2.5 Å	2	0.404	0.174	0.097	0.135	10	0.429	0.180
14 ppm x 14 ppm x 2.5 Å	8	0.286	0.158	0.073	0.094	10	0.315	0.186
16 ppm x 16 ppm x 2.5 Å	7	0.244	0.133	0.057	0.076	10	0.339	0.167
18 ppm x 18 ppm x 2.5 Å	3	0.282	0.173	0.081	0.092	10	0.293	0.184
20 ppm x 20 ppm x 2.5 Å	1	0.397	0.176	0.137	0.152	10	0.358	0.176

*indicates the best PLS and KNN models.

From Table 3 and Figure 4 one can see that the PLS and KNN models achieve their optimal performance at a different granularity of the grid. The best performing PLS model reaches its highest average R^2^_test_ of 0.633 (σ = 0.147) at *10 ppm x 10 ppm x 0.5 Å*, while the KNN model achieves it highest average R^2^_test_ of 0.618 (σ = 0.170) at *2 ppm x 2 ppm x 0.5 Å*. This observation can be explained by the combined effect of several contributing factors:

i) As described in the methodology section, the PLS algorithm utilizes data standardization, which adjusts for the size disparity of variables. Unlike PLS, KNN uses the original bin occupancies. Thus, in the case of PLS, the optimal bin size would mainly reflect the inherent estimation error in the chemical shifts of carbon atoms and their associated inter-atomic distances. As demonstrated in [8], bins with a high resolution on the *Z* axis (C_i_C_j_ distance) and a granularity of at least twice the estimation error of ^13^C chemical shifts in the *XY* plane would result in PLS models of optimal performance. For the current dataset, the ^13^C chemical shifts estimation error was 3.98 ppm, which would require bins with granularity of at least 8 ppm in the *XY* plane. Hence, it is not surprising that the best performing PLS model utilizes *10 ppm x 10 ppm* bins in the *XY* plane and a *0.5 Å* on the *Z*-axis. Besides the ^13^C chemical shifts estimation error, the optimal grid granularity also depends on the bin occupancy. Bins that are too narrow will result in a large, but sparsely populated 3D-SDAR matrix and PLS models unable to generalize (poor predictive performance). On the other hand, models using bins that are too wide (e.g., > 14 ppm in the chemical shifts plane *XY*) may assign fingerprint elements encoding divergent structural features to the same bin, thus producing models lacking in their ability to decode the underlying relationship between structure and activity.

ii) The use of *T* as a factor for activity determination in KNN results in smaller optimal bin sizes in part due to the cancelation of the error in the chemical shifts plane (*XY*) for similar compounds. Note that the highest contribution to the determination of activity in KNN comes only from the first *K*-nearest neighbors, which by definition are most similar to the compound the activity of which is being predicted. Because for similar structures the error of prediction propagates in parallel, it is not surprising that similarity based KNN algorithms will achieve maximum performance at smaller bin sizes.

iii) Unlike PLS, which assigns a different contribution of each bin to the final model, KNN treats all bins as independent coordinates of a vector compared against other such vectors (i.e., assigns equal contribution). Thus, depending on the model building technique being employed, grids of different granularity may be identified as performing better.

### Composite and consensus models

**Figure 5:**
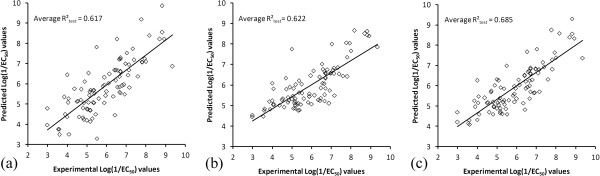
**Plot of the predicted vs. observed log(1/EC**_**50**_**) values in case of: a) the composite PLS model using *10 ppm x 10 ppm x 0.5 Å*** **bins and 7LVs; b) the composite KNN model using *2 ppm x 2 ppm x 0.5 Å *** **bins and 6 neighbors; and c) the PLS-KNN consensus model.**

For both PLS and KNN composite models, Figures 5a and 5b show plots of the average predicted activities of all 94 compounds (each was part of the hold-out test and predicted ~20 times) against their experimental log(1/EC_50_) values. Note that the coefficients of determination shown on Figures 5a and 5b differ slightly from the R^2^_test_ values given in Table 3, as the latter represent an average of 100 individual R^2^_test_ values for the randomized training/test subset pairs.

Because consensus requires at least two individual models an obvious choice was to select complementary composite PLS and KNN models (indicated by the green arrows in Figure 4). This choice allowed the construction of consensus models from pairwise averaging of the predictions from individual models using: i) the same algorithm but different bin size (IDs 2 and 4); ii) the same bin size but different algorithm (IDs 5 and 6) or iii) different algorithm and different bin size (IDs 1 and 3). The percentage improvement in consensus modeling over the average of the coefficients of determination of the individual models is carried out in Table 4. Although consensus between other pairs of individual models or of higher order (averaging predictions from more than two individual models) is possible and may perform better, the enormous number of such models was prohibitive and such efforts were not undertaken.

**Table 4:** **Improvement of R**^**2**^_**test **_**of consensus models over the average R**^**2**^_**test **_**of the individual models (in %)**

**ID**	**Model 1**	**Model 2**	**R**^**2**^_**test**_**for the consensus model**	**Average R**^**2**^_**test **_**of the individual models**	**% improvement**
1	PLS 10 ppm x 10 ppm x 0.5 Å	KNN 2 ppm x 2 ppm x 0.5 Å	0.685	0.620	10.5
2	PLS 10 ppm x 10 ppm x 0.5 Å	PLS 2 ppm x 2 ppm x 0.5 Å	0.673	0.609	10.5
3	PLS 2 ppm x 2 ppm x 0.5 Å	KNN 10 ppm x 10 ppm x 0.5 Å	0.658	0.603	9.1
4	KNN 2 ppm x 2 ppm x 0.5 Å	KNN 10 ppm x 10 ppm x 0.5 Å	0.654	0.614	6.5
5	PLS 2 ppm x 2 ppm x 0.5 Å	KNN 2 ppm x 2 ppm x 0.5 Å	0.640	0.612	4.6
6	PLS 10 ppm x 10 ppm x 0.5 Å	KNN 10 ppm x 10 ppm x 0.5 Å	0.633	0.611	3.6

As can be seen from Table 4, both differences in the data processing algorithms and the granularity of the 3D-SDAR space contribute to the improvement in consensus modeling. A comparison of the performance improvement of consensus models indicates that generally the models of type iii perform best. A possible explanation for this observation is that these models benefit from: i) the complementary information extracted from 3D-SDAR matrices of different granularity and ii) the utilization of different data processing algorithms. Among these 6 consensus models, the one averaging the predictions from the best performing PLS (*10 ppm x 10 ppm x 0.5 Å* bins, 7 LVs) and KNN (*2 ppm x 2 ppm x 0.5 Å* bins, 6 neighbors) individual models was characterized by the highest coefficient of determination (shown in Figure 5c and the last column of Table 2).

To further understand the factors playing a role in consensus modeling and to explain the observed improvement over the composite PLS and KNN models, analysis based on training/test set pairs of individual models was carried out.

According to our initial hypothesis an improvement in consensus modeling would be observed only if the individual composite models account for complementary information (i.e., explain complementary portions of the variance in the biological data). For this purpose, the behavior of the individual 100 sub-models resulting in the best composite PLS and KNN models was investigated. If for each of the 100 training/test set pairs both algorithms capture almost identical structural information encoded in the 3D-SDAR descriptor pool, the corresponding R^2^_test_ values generated on each cycle should be highly correlated and therefore no improvement in consensus modeling would be observed. In other words, the two algorithms would be somewhat redundant and the consensus R^2^_test_ would be an average of R^2^_test_ for the 100 individual sub-models. It has to be emphasized that such an experiment would be valid only in a case of matching training/test subset pairs. This condition is satisfied by the use of the same random seed for both PLS and KNN and a random number generator which was initialized after 100 runs.

**Figure 6:**
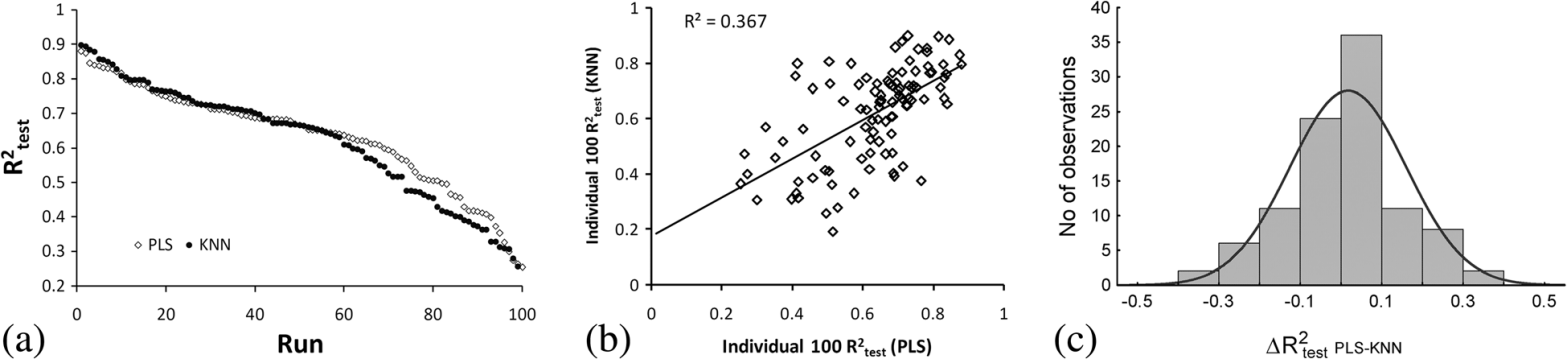
**Ranked (6a) and matched test set pairs (6b) hold-out R**^**2**^_**test **_**of the 100 individual PLS and KNN models producing the best composite models. The distribution of the hold-out R**^**2**^_**PLS**_**-R**^**2**^_**KNN**_**is shown in 6c.**

Three different views of the 100 individual R^2^_test_ values for the best composite PLS (*10 ppm x 10 ppm x 0.5 Å* bins, 7 LVs) and KNN models (*2 ppm x 2 ppm x 0.5 Å* bins, 6 neighbors), are shown in Figure 6. A plot of the ranked R^2^_test_ values for each of these 100 models (Figure 6a) indicated a similar level of performance of both algorithms. Figure 6a also demonstrates that some combinations of training/test subset pairs may produce highly accurate models with R^2^_test_ reaching 0.9, while others may result in models with inferior performance (i.e., models in which the test set compounds are not well represented by the training set).

Figure 6b shows a plot of R^2^_test_ of matching training/test subset pairs processed alternatively by PLS or KNN. Although, there were PLS and KNN sub-models performing equally well (forming a cluster in the upper right corner or the plot), a significant portion of sub-models predicted well by PLS were combined with inferior KNN models and vice-versa. This observation and the relatively low R^2^ of 0.367 suggest that the two individual models reflect different structural patterns in the data and are partially “orthogonal”. The distribution of ΔR^2^_test_ PLS-KNN shown on Figure 6c indicated that a total of 28 models deviate by at least 1σ from the mean. PLS outperformed KNN for 13 models while KNN performed better for the remaining 15 models. These 28 models, for which one of the algorithms succeeded in establishing a structure-activity relationship undetected by the other, were identified as a major contributing factor affecting the performance of consensus models. Thus, a consensus PLS-KNN model would benefit from the partial orthogonality of the PLS and KNN approaches on different sized bins and would outperform the individual composite models.

### Interpretation

**Figure 7:**
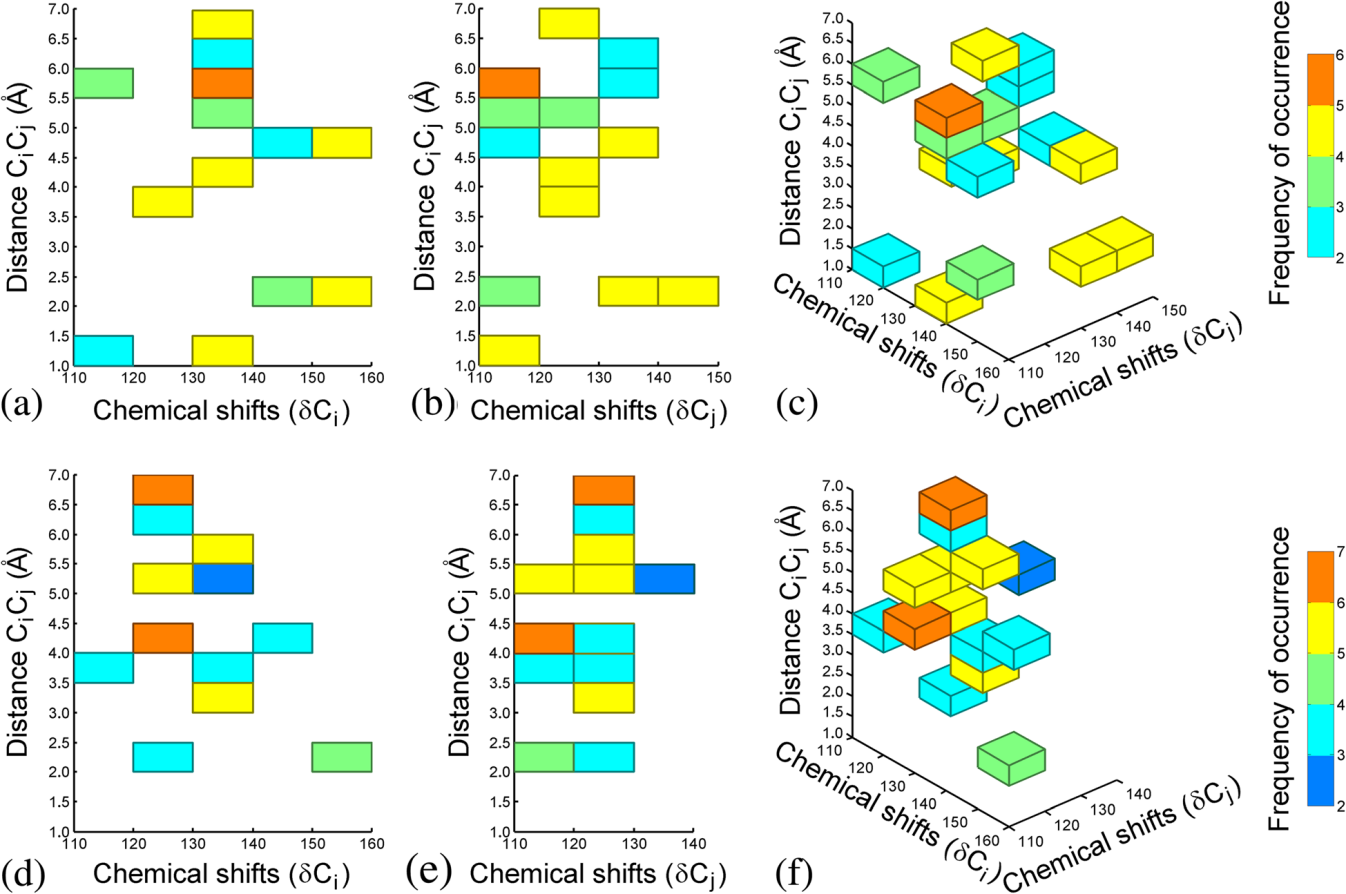
**Orthographic projections in the planes *XZ *(Figures** 7**a and**7**d) and *YZ *(Figures** 7**b and**7**e) of the most frequently occurring bins with positive and negative PLS weights mapped back to the 3D-QSDAR abstract space shown on Figures**7**c and**7**f.**

An essential part of the QSAR modeling process is the interpretation of the model in terms of structural variations leading to corresponding changes in the biological activity. For the purpose of interpretation the bins with the 10 most positive and negative PLS weights for each of the individual models forming the composite *10 ppm x 10 ppm x 0.5 Å* PLS model were extracted and their relative frequencies of occurrence were calculated. Since each of the individual models utilized 7 LVs, a total number of 14000 positive and negative bins were extracted (2 × 100 sub-models × 7 LVs × 10 bins). Unique among these were 87 bins with positive weights and 74 bins with negative weights. Their corresponding relative frequencies of occurrence were calculated and ranked. For simplicity, only the topmost 20% unique positive and negative bins were mapped back to the 3D-SDAR abstract space (see Figure 7).

A detailed examination of the 3D-SDAR maps shown in Figure 7 reveals that none of the bins with positive weights overlaps with any of the bins with negative weights: i.e., the structural features affecting binding (increasing or decreasing log(1/EC_50_)) are well separated. Therefore, compounds with 3D-SDAR fingerprints predominantly occupying bins with positive PLS weights will be stronger binders (highly toxic). Conversely, chemicals with fingerprint elements falling into regions of the 3D-SDAR space occupied by bins with negative weights will be weaker binders (less toxic). This hypothesis was tested using an in house program projecting some of the most frequently occurring positively and negatively weighted bins on the standard coordinate space. This projection allowed identification of subsets of structures in which these bins can often be found together.

**Figure 8:**
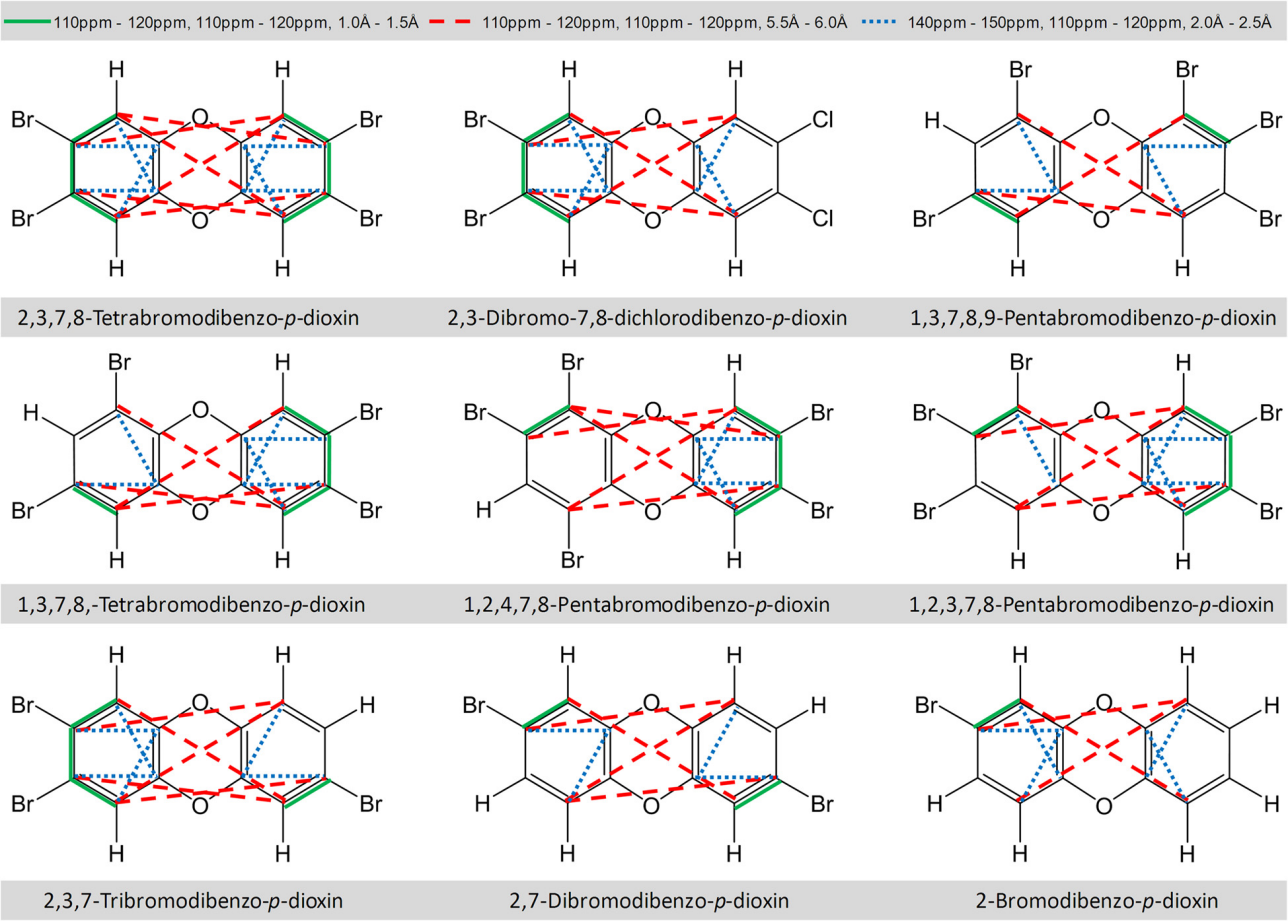
**Frequently occurring positively weighted bins from Figure**6**c superimposed over the structures of dioxins.** For clarity only a few bins are shown, though many more were present.

As was expected, most of the bins characterized by positive PLS weights were found in the structures of PHDDs representing the most toxic class of chemicals in the dataset investigated (see Figure 8). Specifically, a subclass of polybrominated dioxins showed consistent presence of multiple positively weighted bins (see Figure 8). As anticipated, 7 of these were among the top 10 most toxic compounds in the dataset. However, infrequently occurring bins specific to compounds with peculiar structural features did not appear as highly ranked in the composite 3D-QSDAR models. This explains the absence of bins specific to the 2,8-dibromo-3,7-dichlorodibenzo-*p*-dioxin, since it is the only dioxin derivative with both Cl and Br substituents on the same ring.

**Figure 9:**
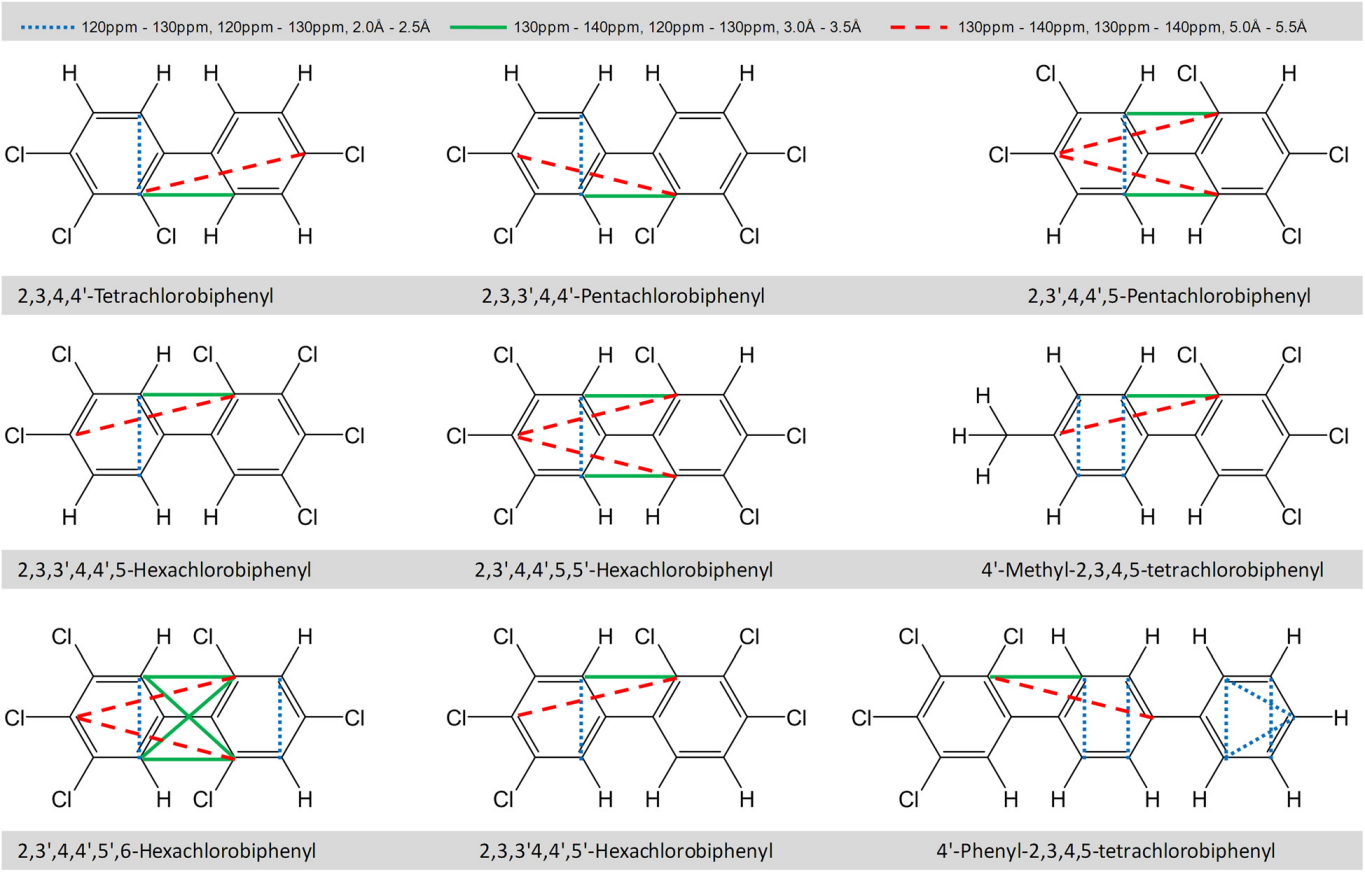
**Frequently occurring negatively weighted bins from Figure **6**f superimposed over the structures of PCBs.** For clarity only a few bins are shown, though more were.

Although in its current version 3D-QSDAR “sees” only the carbon atoms, inferences about their chemical environments can be easily drawn. For example Figure 9 shows that the *140 ppm - 150 ppm, 110 ppm - 120 ppm, 2.0 Å - 2.5 Å* bin is persistently occupied by carbon atoms neighboring the oxygen atoms in PHDDs, indicating the importance of oxygen atoms for binding to AhR [14,31]. Hence, the lack of oxygens in the structure of PCBs can be correlated to their weaker binding affinity (and consequently their lower molar toxicity).

In contrast, most of the negatively weighted bins were found to be present in the structures of PCBs. As can be seen from Figure 9, positions 2 and 2′ and (due to symmetry) positions 6 and 6′ are particularly affected and chlorine substitution at these positions will lower the toxicity of PCBs, compared to that of other chlorine substituted homologues.

As an intermediate chemical class with an average activity higher than that of PCBs and lower than that of PHDDs, the activity of dibenzofurans is affected by the presence/absence of structural patterns similar to those observed in the structures of both PCBs and PHDDs. For example, the presence of an oxygen atom resulting in a chemical shift range of the neighboring carbon atoms between 150 and 160 ppm will lower the EC_50_ of PCDFs (higher toxicity). Analogously to the 2 and 2′ positions in biphenyls, chlorine substitution at positions 1 and 9 will result in PCDFs with toxicity lower than that of PCDF homologues substituted elsewhere.

### Comparison to earlier models

Due to variability in the datasets and the multitude of available data processing algorithms and validation techniques, a direct quantitative comparison with the QSARs summarized in Table 1 is impossible. However, if one takes into account the much stringent validation criteria imposed in our work (vs the cross-validation procedures employed in [13-21]) it is clear that the 3D-QSDAR methodology performs at least on par with these earlier models. Similarly to CoMFA [17] on a qualitative level the 3D-QSDAR was able to recognize correctly the positions that affect the strength of binding to AhR. Since our work is based on a dataset originally compiled by Mekenyan et al. a more direct comparison with the QSARs reported in [22] was possible. Multiple separate QSARs for the three classes of PCBs, PHDDs and PCDFs with R^2^ ranging from 0.640 (*n* = 30) to 0.899 (*n* = 14) were derived. The statistical parameters of a model combining the most planar PCBs, PHDDs and PCDFs (*n* = 80) were as follows: R^2^ = 0.73; s^2^ = 0.59; R^2^_cv_ = 0.73 and F = 69.2. In comparison, for the complete set of 94 compounds our best consensus model produced an R^2^_test_ of 0.685 and a q^2^_LOO_ of 0.79 which are both close to the R^2^_cv_ of 0.73 reported by Mekenyan et al.

## Conclusions

We have introduced several validation techniques intended to improve the quality and reliability of individual and consensus QSAR models. Their use was illustrated on a dataset of 94 AhR binders modeled by 3D-QSDAR. The functional dependence between R^2^_test_ and the number of training/test subset randomization cycles was used to determine the minimum number of cycles necessary to achieve convergence of R^2^_test_ to its asymptotic “true” value. In this specific case, which uses 20% of the compounds as a hold-out test set, 100 randomization cycles proved sufficient for achieving convergence for both PLS and KNN models. The use of a distance measure (Tanimoto similarity) as a discriminant function in KNN was shown to produce models with performance similar to that of PLS when applied to the same dataset. A plot of R^2^_test_ for matching test set pairs was used to demonstrate the partial orthogonality of PLS and similarity based KNN approaches on different bin granularity. However, further investigations may shed additional light on the character of the multiple factors playing role in the improvements observed in consensus modeling.

In the last stage of the modeling process the most frequently occurring positively and negatively weighted bins were projected back to the standard coordinate space to identify structural features related to toxicity. It was found that most of the highly ranked bins with positive PLS weights were specific to a class of polybrominated dioxins. The oxygen atoms of PHDDs and PCDFs participating in formation of donor-acceptor bonds with the receptor were associated with the high toxic effect of these two chemical classes. In the absence of other substituents, PCBs with chlorine atoms at positions 2 and 2′ (and due to symmetry positions 6 and 6′) were accurately predicted to be relatively weaker binders (less toxic).

## Abbreviations

PLS: Partial Least Squares; KNN: k Nearest Neighbors; 3D-SDAR: Three-Dimensional Spectral Data - Activity Relationship; AhR: Aryl Hydrocarbon Receptor; PCBs: Polychlorinated Biphenyls; PHDDs: Polyhalogenated Dibenzo-p-Dioxins; PCDFs: Polychlorinated Dibenzofurans; QSAR: Quantitative Structure - Activity relationship; LOO: Leave-One-Out; CV: Cross-Validation; MLR: Multiple Linear Regression.

## Competing interests

The authors declare that they have no competing interests.

## Authors' contributions

SS worked on the main concepts, significant portion of the data processing code, the computational experiments design and the data analysis and interpretation; BP wrote the routines for 3D-SDAR space tessellation into bins; DB carried out experiments using non-linear pattern recognition methods to confirm that narrow bins could be used to improve consensus model predictions in combination with other pattern recognition methods using larger bins (unpublished but conceptually important data); RB conceived various experimental designs addressing questions regarding the factors playing role in consensus modeling, and participated in all discussions concerning the improvements in the 3D-QSDAR methodology; JW proposed different hypotheses explaining the observed improvements in consensus modeling and proposed new experiments that would quantify the impact of each contributing factor. All authors read and approved the final manuscript.

## Supplementary Material

Additional file 1 Matlab code used for generation of the randomized hold-out test sets. Click here for file
