# Supplementary material for: Complementary PLS and KNN algorithms for improved 3D-QSDAR consensus modeling of AhR binding
Source: J Cheminform. 2013 Nov 21;5:47. doi: 10.1186/1758-2946-5-47 (PMC3843526; doi:10.1186/1758-2946-5-47)
Supplement: Additional file 1 — Matlab code used for generation of the randomized hold-out test sets. [file 1758-2946-5-47-S1.doc]

% Initialization of the random number generator

s=RandStream('mt19937ar','seed',0);

RandStream.setDefaultStream(s);

for ValidationCycles=1:cycles

% Definition of the training and test subsets

[Training, Test] = crossvalind('HoldOut', samples, TstRatio);

IDTR=ID(Training==1); IDTS=ID(Test==1);

ATR=ACT(Training==1); ATS=ACT(Test==1);

MTR=MAT(Training==1,:); MTS=MAT(Test==1,:);

%PLS or KNN code goes here

end
